# Efficacy of a novel topical combination of esafoxolaner, eprinomectin and praziquantel for the prevention of heartworm disease in cats

**DOI:** 10.1051/parasite/2021026

**Published:** 2021-04-02

**Authors:** Christine Baker, John McCall, Abdelmoneim Mansour, Scott McCall, Tayna Shaffer, Kenneth Wakeland, Elizabeth Mitchell, Justin Frost, Eric Tielemans, Dwight Bowman

**Affiliations:** 1 Boehringer-Ingelheim Animal Health 1730 Olympic Drive Athens 30601 GA USA; 2 TRS Labs Inc. 215 Paradise Blvd Athens 30607-1151 GA USA; 3 ClinVet USA 1479 Talmadge Hill Rd S Waverly 14892 NY USA; 4 Boehringer-Ingelheim Animal Health 29 Avenue Tony Garnier 69007 Lyon France; 5 College of Veterinary Medicine, Cornell University Ithaca 14850 NY USA

**Keywords:** Cat, Heartworm disease, *Dirofilaria immitis*, Eprinomectin, Efficacy

## Abstract

NexGard^®^ Combo is a novel topical endectoparasiticide formulation for cats combining the insecticide/acaricide esafoxolaner, the nematodicide eprinomectin and the cestodicide praziquantel. The efficacy of this novel formulation for the prevention of heartworm disease in cats was tested in two experimental studies using an induced infection model and a randomized, blinded, placebo-controlled study design, and two USA isolates of *Dirofilaria immitis*. In each study, 20 naïve cats were each inoculated sub-cutaneously with 100 third-stage larvae of *D. immitis* 30 days before treatment. Following randomization to two treatment groups of ten cats, each cat was treated topically once, either with the minimum recommended dose of the novel formulation, or with an identical volume of placebo. Five months after treatment (6 months after infections), the cats were humanely euthanized for parasite recovery and count. Efficacy was calculated by comparison of the numbers of adult *D. immitis* recovered in the control and in the novel formulation groups. In the control groups of each study, *D. immitis* were recovered in seven and nine cats (respective worm counts ranges 1–7 and 1–16, respective geometric means 1.6 and 5.1). In both studies, none of the treated cats harbored any *D. immitis* at necropsy and the calculated efficacy of the novel formulation was 100%. There were no adverse reactions related to treatment with the novel formulation. The results of these two studies demonstrate that a topical NexGard^®^ Combo application at the minimum label dose is well-tolerated and efficacious in preventing heartworm disease in cats.

## Abstract (fr)

NexGard^®^ Combo est une nouvelle formulation d’endectoparasiticide topique pour chats combinant l’insecticide/acaricide esafoxolaner, le nématodicide éprinomectine et le cestodicide praziquantel. L’efficacité de cette nouvelle formulation pour la prévention de la maladie du ver du cœur (dirofilariose) chez les chats a été testée dans deux études expérimentales utilisant un modèle d’infection induite et une conception d’étude randomisée, en aveugle et contrôlée par placebo, et deux isolats américains de *Dirofilaria immitis*. Dans chaque étude, vingt chats naïfs ont chacun été inoculés par voie sous-cutanée avec 100 larves de troisième stade de *D. immitis* 30 jours avant le traitement. Après randomisation dans deux groupes de traitement de dix chats, chaque chat a été traité par voie topique une fois, soit avec la dose minimale recommandée de la nouvelle formulation, soit avec un volume identique de placebo. Cinq mois après le traitement (6 mois après les infections), les chats ont été euthanasiés sans cruauté pour la récupération et le dénombrement des parasites. L’efficacité a été calculée en comparant les nombres de *D. immitis* adultes collectés dans le groupe contrôle et dans le groupe ayant reçu la nouvelle formulation. Dans les groupes témoins de chaque étude, *D. immitis* a été trouvé chez sept et neuf chats (les nombres de vers respectifs variaient de 1 à 7 et de 1 à 16, les moyennes géométriques respectives étaient 1,6 et 5,1). Dans les deux études, aucun des chats traités ne présentait de *D. immitis* lors de l’autopsie et l’efficacité calculée de la nouvelle formulation était de 100%. Il n’y a eu aucun effet indésirable lié au traitement avec la nouvelle formulation. Les résultats de ces deux études démontrent qu’une application topique de NexGard^®^ Combo à la dose minimale indiquée sur l’étiquette est bien tolérée et efficace pour prévenir la dirofilariose chez les chats.

## Introduction

The filarial nematode *Dirofilaria immitis* is the agent of heartworm disease (dirofilariosis), a serious, untreatable, and sometimes fatal disease in cats. Wild and domestic canid species are the natural definitive hosts and reservoirs for *D. immitis*, while other carnivores, such as felines and mustelidae can also be infected and may serve as reservoirs during the short period that they are microfilaremic. Humans rarely become accidental hosts to *D. immitis,* mostly with asymptomatic pulmonary infarction, embolism and nodule formation incidentally detected on chest radiographs [27, 31].

The obligate intermediate hosts are mosquitoes of several genera, including *Aedes, Culex,* and *Anopheles* [4–6, 21]*. Dirofilaria immitis* is distributed worldwide and transmitted by mosquitoes year round in warm and humid climates (tropical and sub-tropical), or seasonally in temperate regions including most of the United States, Canada, Japan, areas of Australia, and Southern Europe. Local prevalence significantly increases when high density of reservoir species and intermediate host populations coexist and perpetuate the lifecycle [7–9, 12, 13, 15–18, 20, 23, 24, 30, 32, 34].

Mosquitoes are infected by *D. immitis* during blood meals on *D. immitis* microfilaraemic reservoir hosts. In the mosquitoes, the microfilariae grow into infective third-stage larvae of *D. immitis* (L_3_) in approximately 10–14 days in optimum conditions. Then, mosquitoes infect mammalian hosts with L_3s_ during blood meals. After a few days in the subcutaneous tissue of the host, the L_3_ molts into a L_4_ that starts migrating through the body. Two to three months after infection, the surviving L_4s_ molt into immature adults that access the venous system and may reach the right heart cavity and pulmonary arteries, 2–4 months after infection. There, the larvae fully mature into an adult, about 6–9 months after infection.

Typical clinical signs of feline heartworm disease manifest as a cardiopulmonary syndrome called Heartworm-Associated Respiratory Disease (HARD), which may progress in two phases [10, 11, 35, 36]. Initially, about 4–6 months after infection, there typically is an acute inflammatory reaction to *D. immitis* immature adults as they reach the pulmonary arteries. This may be asymptomatic or cause acute or chronic cardiopulmonary signs. Signs of gastrointestinal or nervous system involvement are less frequent but may result from severe inflammation, immune reaction, or ectopic migration of the parasite stages [19]. Many of the immature stages are eliminated during this first phase but chronic lesions may persist with associated histological abnormalities and clinical signs in a significant proportion of cats, even after the death of *D. immitis* larval and immature stages [5, 22]. The second phase occurs when the surviving adult *D. immitis* established in the pulmonary arteries and right heart chambers cause inflammation, resulting in pathology and clinical signs similar to canine dirofilariosis. In comparison to dogs, cats usually harbor fewer mature heartworms (typically 1–4) and these worms have a shorter lifespan (usually 1–2 years). Nevertheless, any adult heartworm burden tends to be clinically more significant in cats than in dogs, due to their strong inflammatory reaction to the presence of live worm and worm death [10, 11].

Gross necropsy surveys conducted over the past 50 years in feral cats in enzootic regions have revealed a prevalence of heartworm lesions ranging from 0.5% to 14% [9]. The geographic distribution of the parasite in cats mirrors that of dogs, with an estimated 5–10 times lower prevalence than that of the canine population [1, 3, 18, 21]. However, the true prevalence of *D. immitis* infection in cats is probably underestimated due to less frequent testing and to a high incidence of false-negative laboratory tests. As they target heartworm antigen, antibody or microfilariae, these tests are best suited to detect long-lasting patent infections with high numbers of mature heartworms, as occurs in canine heartworm infections. Tests are mostly negative when only immature *D. immitis* are present, as in the first phase of the feline infection. The detection of adult *D. immitis* in cats with clinical signs of heartworm disease is improved when a combination of tests is employed, although all tests may be falsely negative, especially when there is no adult female worm or when a single adult worm is present, as is frequent in feline heartworm disease [3, 5, 22, 28, 33, 36].

NexGard^®^ Combo is a novel topical formulation designed to eliminate or prevent the development of numerous endo and ectoparasites in cats through the combination of three active ingredients: esafoxolaner, eprinomectin, and praziquantel. Eprinomectin (an avermectin of the macrocyclic lactone class) kills larvae of various nematode parasites including *D. immitis*. Broadline^®^ (Boehringer-Ingelheim), a topical endectoparasiticide, and Centragard^®^ (Boehringer-Ingelheim), a topical endoparasiticide, are used in many parts of the world to prevent heartworm disease in cats. Both of these formulations use the same eprinomectin dosage as in NexGard^®^ Combo [2]. This manuscript reports two studies of the efficacy of NexGard^®^ Combo to prevent heartworm disease in cats, using two recent isolates of *D. immitis* in an induced laboratory model of infection, as required to demonstrate the efficacy of eprinomectin in this novel formulation and for regulatory approval.

## Materials and methods

### Ethics

Cats were managed with due regard for their wellbeing and the study designs were reviewed and approved by the Sponsor and local Institutional Animal Care and Use Committees.

### Study designs

Both studies followed a randomized, blinded, and placebo-controlled design and were conducted in compliance with the International Cooperation on Harmonisation of Technical Requirements for Registration of Veterinary Medicinal Products (VICH) VICH GL9 “Good Clinical Practice”, and were designed in general accordance with the local regulatory requirements, the VICH GL7 “Efficacy of Anthelmintics: General Requirements”, the VICH GL20 “Efficacy of Anthelmintics: Specific Recommendations for Felines”, and the “World Association for the Advancement of Veterinary Parasitology (WAAVP) guidelines for evaluating the efficacy of anthelmintics for dogs and cats.”

### Animals and health

All cats were healthy, purpose-bred Domestic Short-hair cats, which had never been treated with macrocyclic lactones, and were born and raised in mosquito-protected environments at a licensed breeder facility. Cats were acclimated to each study environment for at least seven days before *D. immitis* infection and were housed in an environmentally-controlled mosquito proof facility until the end of the study. Table 1 lists the body weight, age, and sex of the cats.

**Table 1:** Heartworm isolates, study locations and animal details.

Study	Isolate of *D. immitis*	Study location	Animals		
			Sex	Bodyweight at treatment (kg)	Age at Infection (months)
Study 1	Berkeley^a^	Waverly NY, USA	10 males, 10 females	2.4–5.5	6.1–6.4
Study 2	Georgia II^b^	Athens GA, USA	10 males, 10 females	2.0–3.0	3.5–3.7

a The Berkeley isolate was obtained in Berkeley County (South Carolina, USA) in April 2014 and maintained in laboratory conditions with three passages since validation in December 2014 at TRS Labs.

b The Georgia II isolate was obtained in Vidalia (Georgia, USA) in April 2013 and maintained in Laboratory conditions with one passage since validation in December 2013 at TRS Labs.

After an initial veterinary examination before *D. immitis* infection, all cats were observed to detect any health abnormality at least once daily throughout the study, and hourly four times after treatment.

Although the cats had been raised in mosquito-free environments, the lack of evidence of infection prior to the study’s L3 inoculations was verified by *D. immitis* antigen tests (ELISA DiroCHECK^®^) conducted on Day 90, i.e. at four months after the L3 inoculations.

### Laboratory model of induced *D. immitis* infection

Two recent *D. immitis* isolates were used at different research laboratories (Table 1). The *D. immitis* inoculum was prepared and administered in an identical manner in both studies. First, *Aedes aegypti* mosquitoes were fed on heparinized blood containing *D. immitis* microfilariae on a membrane feeding device, 15–16 days prior to inoculation. On the day of inoculation (Day −30), *D. immitis* L_3s_ were harvested from these mosquitoes into Hanks balanced salt solution (HBSS). For infection of each cat, 100 L_3s_ were counted using a dissecting microscope, drawn with a small amount of HBSS into a 1 mL tuberculin syringe, and injected subcutaneously in the inguinal area of the cat shortly thereafter.

### Treatment

Ten cats per group were treated with a placebo or with NexGard^®^ Combo once on Day 0. For each treatment, the appropriate product was applied directly on the skin after parting the hair on one spot in the midline of the neck between the base of the skull and the shoulder blades. Cats assigned to the NexGard^®^ Combo groups were treated at the minimum recommended dose of 0.12 mL/kg, providing 1.44 mg/kg esafoxolaner, 0.48 mg/kg eprinomectin, and 10.0 mg/kg praziquantel. Cats assigned to the placebo control group were treated with the same volume (0.12 mL/kg) of mineral oil.

### Necropsy and adult *D. immitis* worm counts

All procedures were conducted by blinded personnel. Cats were humanely euthanized for heartworm counts six months after infections, except for one control cat in Study 1, which was humanely euthanized for necropsy on study Day 69 due to urethral blocage. To detect and count all heartworms at necropsy, the pleural and peritoneal cavities of all cats were opened and carefully explored. The heart was dissected and the right ventricle and atrium were examined for worms. The pulmonary arteries were dissected through their distal branches in each lung lobe, as were the venae cavae and major hepatic veins. All heartworms and heartworm fragments were collected, identified as male or female *D. immitis*, and counted.

### Statistical analysis

The total counts of live *D. immitis* were transformed to the natural logarithm of (count +1) to calculate the geometric means for each treatment group. The efficacy of the novel formulation against *D. immitis* was determined by calculating the percent efficacy as 100 × ([*C* – *T*]/*C*), where *C* was the geometric mean among control animals, and T was the geometric mean among animals treated with the novel formulation. The log-counts of the animals treated with the novel formulation were compared to the log-counts of the control animals using an *F*-test adjusted for the allocation blocks (animal sex) used to randomize the animals to the treatment groups. The MIXED procedure in SAS was used for the analysis, with the treatment group listed as a fixed effect and the animal sex listed as a random effect. All testing was two-sided at the significance level (*α*) of 0.05.

## Results

### Antigen tests

In both studies, all cats had negative *D. immitis* antigen ELISA tests at four months after the induced infections.

### Efficacy

The heartworm counts are presented in Table 2. A total of 75 and 25 live heartworms were recovered, from nine and seven of the ten control cats in Studies 1 and 2, respectively while all cats treated with NexGard^®^ Combo had no heartworms. In both studies, the calculated efficacy of NexGard^®^ Combo was 100% for the prevention of feline heartworm disease. In addition, the heartworm counts were significantly different between the novel formulation and the placebo-treated groups (*p* < 0.0001 and *p* = 0.0024, respectively in Studies 1 and 2). Additionally, it was observed in Study 2 that all cats with one or more heartworms also had grossly visible thickening of the pulmonary arteries, which was suggestive of heartworm disease (histopathology was not performed).

**Table 2:** Heartworm count per treatment group and efficacy of NexGard^®^ Combo against *D. immitis* in cats.

	Treatment groups^a^	*n*	Total *D. immitis* counts in individual cats at necropsy^b^	*D. immitis* counts geometric mean	Efficacy	*P*-value	Total live *D. immitis* counts by sex/Total live count ranges per cat by sex
Study 1	Control (Placebo)	10	0, 1, 2, 5, 8, 8, 8, 14, 16, 16	5.1	NA	NA	32 m & 43 f/2–9 m & 1–11 f
	NexGard^®^ Combo	10	0 in each of the 10 cats	0.0	100%	<0.0001	0/0
Study 2	Control (Placebo)	10	0, 0, 0, 1, 1, 1, 3, 6, 6, 7	1.6	NA	NA	15 m & 10 f/1–4 m & 1–4 f
	NexGard^®^ Combo	10	0 in each of the 10 cats	0.0	100%	0.0024	0/0

m = male; f = female; NA = not applicable.

a Placebo group treated once on Day 0 with 0.12 mL/kg of mineral oil and NexGard^®^ Combo Group treated once on Day 0 with 0.12 mL/kg of the novel formulation delivering 1.44 mg/kg esafoxolaner, 0.48 mg/kg eprinomectin, and 10.0 mg/kg praziquantel.

b All *D. immitis* were alive, except in two cats in Study 1 having one or 2 dead fragmented *D. immitis* out of 8 and 16, respectively.

### Tolerance

Cats had no health abnormality related to treatment with the novel formulation in either study. Clinical signs of heartworm disease were not observed in any of the cats at any time during the two studies.

## Discussion

As expected in cats maintained in a mosquitoe-proof environment, all cats in both studies had negative *D. immitis* antigen tests at 4 months after infections, showing no evidence of infection prior to the study.

Feline antibody tests were not conducted because they do not provide a definitive diagnosis of heartworm infection, even in cats with clinical signs of heartworm disease [29]. In one study, most antibody tests conducted on experimentally infected control cats were positive by 2 months after infection [25] and in another study, some experimentally infected cats treated with a macrocyclic lactone preventive 1 month after infection remained antibody-positive for a few months, even after larvae had been eliminated [26].

At necropsy, the Study 1 control cat that was euthanized on Day 69 due to a urinary blockage had 14 small but well-identifiable heartworms recovered from the pulmonary arteries, which were included in the efficacy calculation.

In both studies, one treatment with the minimum recommended dose of NexGard^®^ Combo had comparable efficacy against *D. immitis* as Broadline^®^. Eprinomectin, the active ingredient against *D. immitis* has also been shown to have similar plasma profiles with both combination formulations [14].

As is typical with feline heartworm infections, the number of heartworms recovered was lower than in canine hosts. However, infection with a single heartworm is significant in cats as the magnitude of the inflammatory reaction to a single worm may result in severe and potentially fatal disease [10, 11]. Although some cats may appear to recover spontaneously from natural infections, their inflammatory reaction to one or more adult heartworms, particularly as a consequence of worm death, may cause thromboembolism, severe lung damage, respiratory failure, and death. In a three year study monitoring clinical signs in 34 cats with naturally acquired heartworm disease, 28 cats (82%) “self-cured” (21 without clinical signs), but 6 (18%) died suddenly or with acute respiratory failure [35]. Therefore, if the disease had been left to run its course beyond 6 months after infection in our studies, it is likely that several of the 16 control cats with 1–16 live adult heartworms would have developped potentially deadly heartworm disease. The reason for the overall lower heartworm burden observed in Study 2 compared to Study 1 is unknown. The same expert parasitologist conducted the infection in an identical manner in both studies. The necropsies and worm counts were completed by different but experienced parasitologists. Although all possible precautions were taken to prevent cross-contamination during handling of the control and NexGard^®^ Combo groups, a low level of inadvertent cross-contamination cannot be definitively ruled out. On the other hand, these results might just reflect the high variability of this laboratory model.

The tolerance to the novel formulation was excellent, with no adverse reactions to NexGard^®^ Combo treatment.

In light of the absence of any known adulticidal treatment of feline heartworm disease and of the potential severity of its clinical signs at early stages of infection, chemoprophylaxis is necessary to protect cats living in *D. immitis* endemic areas. NexGard^®^ Combo provides an efficacious, safe, and easy to use monthly chemoprophylaxis for the prevention of heartworm disease in cats.

## Competing interest

The work reported herein was funded by Boehringer-Ingelheim and some of the authors are employees of Boehringer-Ingelheim Animal Health. Other than that, the authors declare no conflict of interest. This document is provided for scientific purposes only. Any reference to a brand or trademark is for information purposes only and is not intended for any commercial purposes or to dilute the rights of the respective owners of the brand(s) or trademark(s). NexGard^®^ is a registered trademark of the Boehringer Ingelheim Group.
